# Interconnectivity between molecular subtypes and tumor stage in colorectal cancer

**DOI:** 10.1186/s12885-020-07316-z

**Published:** 2020-09-04

**Authors:** R. R. J. Coebergh van den Braak, S. ten Hoorn, A. M. Sieuwerts, J. B. Tuynman, M. Smid, S. M. Wilting, J. W. M. Martens, C. J. A. Punt, J. A. Foekens, J. P. Medema, J. N. M. IJzermans, L. Vermeulen

**Affiliations:** 1 Department of Surgery, Erasmus MC University Medical Center, ‘s Gravendijkwal 230, 3015 CE Rotterdam, The Netherlands; 2 Laboratory for Experimental Oncology and Radiobiology, Amsterdam UMC, University of Amsterdam and Cancer Center Amsterdam, Meibergdreef 9, 1105AZ Amsterdam, The Netherlands; 3 Oncode Institute, Amsterdam UMC, Meibergdreef 9, 1105AZ Amsterdam, The Netherlands; 4 Department of Medical Oncology, Erasmus MC Cancer Institute, Erasmus MC University Medical Center, Wytemaweg 80, 3015 CN Rotterdam, The Netherlands; 5 Cancer Genomics Center Netherlands, Amsterdam, The Netherlands; 6 Department of Surgery, Amsterdam UMC, Boelelaan 1117, 1081 HV Amsterdam, The Netherlands; 7 Department of Medical Oncology, Amsterdam UMC, University of Amsterdam, Meibergdreef 9, 1105AZ Amsterdam, The Netherlands; 8 Julius Center for Health Sciences and Primary Care, University Medical Center Utrecht, Universiteitsweg 100, 3584 CX Utrecht, The Netherlands

**Keywords:** Colorectal cancer, Molecular subtype, Tumor biology, CMS, TNM

## Abstract

**Background:**

There are profound individual differences in clinical outcomes between colorectal cancers (CRCs) presenting with identical stage of disease. Molecular stratification, in conjunction with the traditional TNM staging, is a promising way to predict patient outcomes. We investigated the interconnectivity between tumor stage and tumor biology reflected by the Consensus Molecular Subtypes (CMSs) in CRC, and explored the possible value of these insights in patients with stage II colon cancer.

**Methods:**

We performed a retrospective analysis using clinical records and gene expression profiling in a meta-cohort of 1040 CRC patients. The interconnectivity of tumor biology and disease stage was assessed by investigating the association between CMSs and TNM classification. In order to validate the clinical applicability of our findings we employed a meta-cohort of 197 stage II colon cancers.

**Results:**

CMS4 was significantly more prevalent in advanced stages of disease (stage I 9.8% versus stage IV 38.5%, *p* < 0.001). The observed differential gene expression between cancer stages is at least partly explained by the biological differences as reflected by CMS subtypes. Gene signatures for stage III-IV and CMS4 were highly correlated (r = 0.77, *p* < 0.001). CMS4 cancers showed an increased progression rate to more advanced stages (CMS4 compared to CMS2: 1.25, 95% CI: 1.08–1.46). Patients with a CMS4 cancer had worse survival in the high-risk stage II tumors compared to the total stage II cohort (5-year DFS 41.7% versus 100.0%, *p* = 0.008).

**Conclusions:**

Considerable interconnectivity between tumor biology and tumor stage in CRC exists. This implies that the TNM stage, in addition to the stage of progression, might also reflect distinct biological disease entities. These insights can potentially be utilized to optimize identification of high-risk stage II colon cancers.

## Background

Colorectal cancer (CRC) is the fourth most common cancer worldwide and the second leading cause of cancer mortality [1]. Clinical decision making in CRC is mainly driven by clinical and traditional pathological features including TNM staging. Although these features hold considerable prognostic, and even predictive value, there are profound individual differences in clinical outcome within a single tumor stage, especially for stage II and III [2]. Also, there is compelling evidence that not all cancers follow the linear-progression model associating with the TNM-stages. For example, in CRC the majority of lymphatic and distant metastases arise from independent subclones, and 40–63% of metachronous metastases develop in patients without lymph node metastasis [3]. The consensus molecular subtype (CMS) classification is a widely studied transcriptome-based stratification system for CRC defining four disease entities (CMS 1–4) with distinct clinical, biological and molecular features [4]. Hence, the CMS taxonomy could offer a framework to elucidate whether TNM solely resembles disease progression or also biologically different entities that preferentially present with a specific stage of disease at diagnosis. This study was conducted to investigate the interconnectivity between tumor stage and tumor biology in CRC patients. Subsequently we demonstrate the added value of this knowledge in patients with high-risk stage II colon cancer, a subgroup in which accurate prognostication and selection for adjuvant treatment is still an unmet need.

## Methods

### Patients and data aggregation

Patients for which information on staging and microsatellite instability (MSI) status was available were selected from the previously reported meta-cohort of Guinney et al. [4], resulting in 1040 individual patients (accession number GSE39582 [5] and TCGA). For validation of our findings the chemotherapy naïve stage II CRC patients of the MATCH Cohort [6] and AMC-AJCCII-90 Cohort (accession number GSE33113) [7] were used. In the validation cohort high-risk was defined as either T4 or inadequate lymph node assessment (< 10 nodes assessed).

The R2: Genomic Analysis and Visualization Platform was used to extract the aggregated and normalized data (http://r2.amc.nl).

### CMS classification

Samples were classified into molecular subtypes using the Random Forest (RF) method available in the R package of the CMS classifier (v1·0·0, https://www.synapse.org/#!Synapse:syn4961785) [4].

### Differential gene expression analysis

The limma package was used to identify differentially expressed genes (DEG) between the different tumor stages and CMS groups, using the ANOVA test for overall DEG and a limma-test for individual groups. *P*-values were FDR corrected. For comparing the number of DEG between the overall cohort and CMSs, a random set of 200 patients was sampled 1000 times to correct for the effect of group size on the number of DEG.

### Gene signatures

Gene signatures for advanced stage and CMS4 were built using the top 100 DEG (with the lowest FDR corrected *p*-value) between early (stage I-II) and advanced stage (stage III-IV), and CMS1/2/3 and CMS 4. Gene signature scores were built using the weighted matched z-score of both the up- and downregulated genes of the gene signatures.

### Statistical analysis

The Chi-square test was used to assess associations between CMS classification and tumor stage. The Kaplan-Meier method was used to estimate survival. Survival curves were compared using the log-rank test. Disease-free survival (DFS) times of > 60 months were censored at 60 months. We performed a multivariate analysis using a Cox proportional-hazards model with CMS, gender, age, tumor location, T-stage and MSI status as covariables. All statistical tests were 2-sided and considered significant at a *P*-value lower than 0·05. All analyses were performed using R version 3.6.1.

## Results

### Distinct TNM stages represent with different distributions of molecular subtypes

**Fig. 1:**
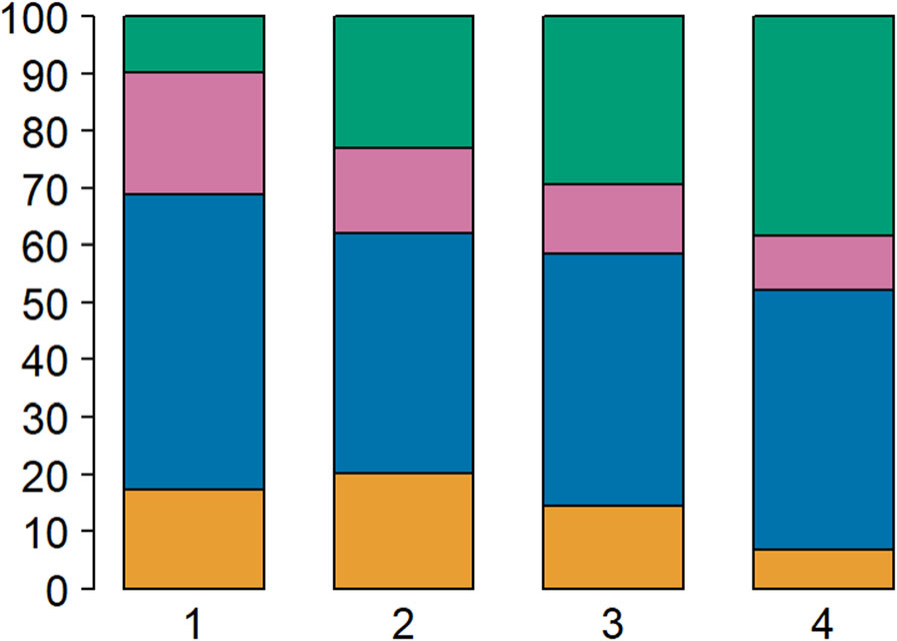
Distribution of the molecular subtypes for each tumor stage. Distribution in percentages (y-axis) of the CMS groups in the cohort

We analyzed the association between CMS subtypes and tumor stage in a meta-cohort comprising 1040 patients (Table 1). An increase in prevalence of the poor-prognosis mesenchymal subtype (CMS4) was detected in advanced stages of disease (stage I 12 (9.8%), stage II 89 (22.9%), stage III 94 (29.4%) and stage IV 45 (38.5%), *p* < 0.001) (Fig. 1 and Additional file 1: Table S1). The same increase was observed for the individual cohorts separately (Additional file 1: Table S1 and Additional file 1: Fig. S1).

**Table 1:** Basic characteristics of the aggregated cohort (*n* = 1040)

		Total		GSE39582		TCGA	
		*n* = 1040		*n* = 511		*n* = 529	
**Gender**	Female	476	45.8%	227	44.4%	249	47.1%
	Male	564	54.2%	284	55.6%	280	52.9%
**Age**	Median (IQR^a^)	68 (59–77)		69 (59–76)		68 (59–77)	
**TNM**	I	133	12.8%	38	7.4%	95	18.0%
	II	417	40.1%	216	42.3%	201	38.0%
	III	355	34.1%	200	39.1%	155	29.3%
	IV	135	13.0%	57	11.2%	78	14.7%
**MSI**	MSS	887	85.3%	436	85.3%	451	85.3%
	MSI	153	14.7%	75	14.7%	78	14.7%
**CMS**	1	153	14.7%	79	15.5%	74	14.0%
	2	420	40.4%	214	41.9%	206	38.9%
	3	133	12.8%	66	12.9%	67	12.7%
	4	240	23.1%	112	21.9%	128	24.2%
	Indeterminate	94	9.0%	40	7.8%	54	10.2%

^a^*IQR* Interquartile range

### Tumor stage reflects tumor biology

**Fig. 2:**
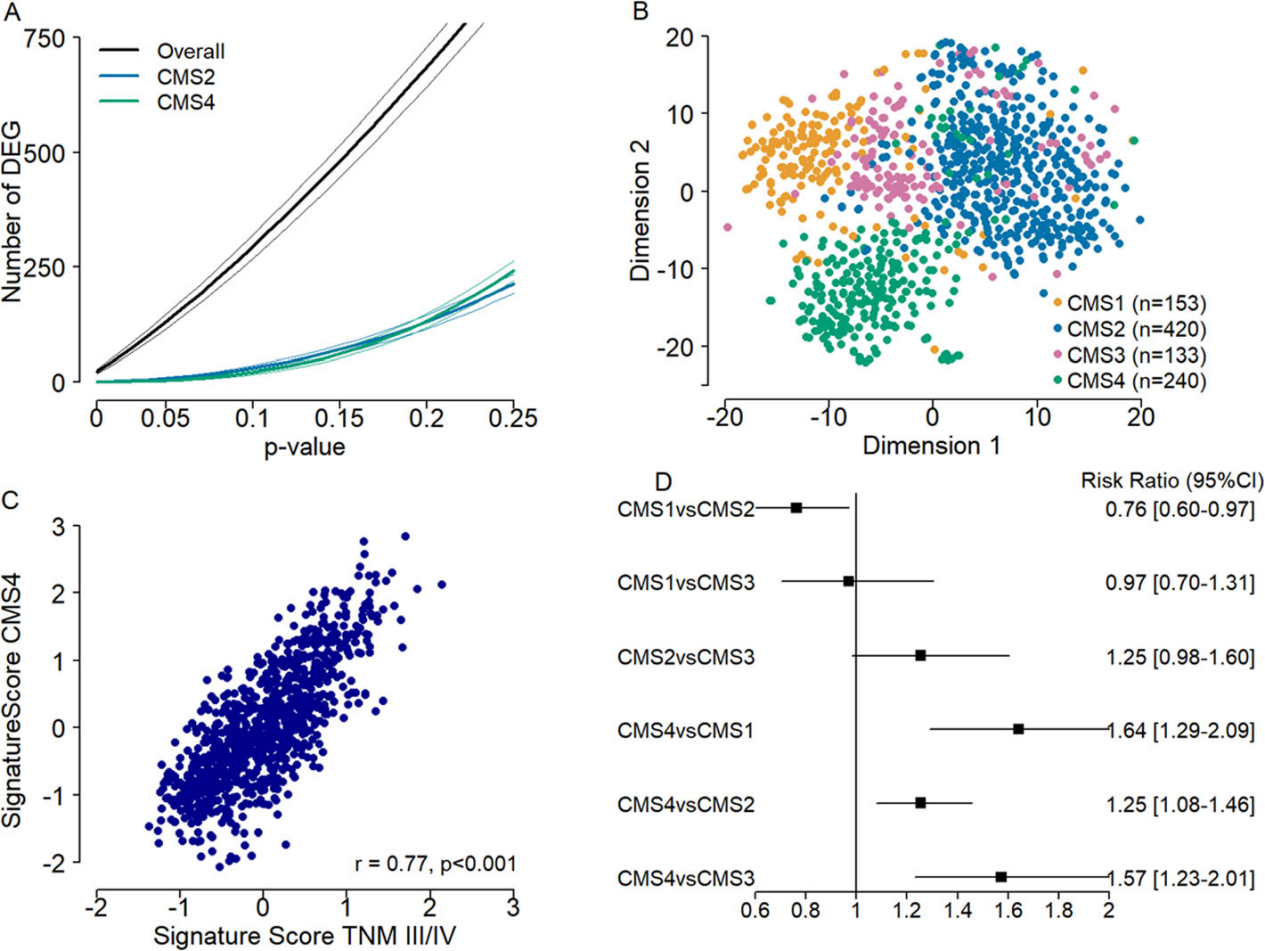
Gene expression analysis and risk ratio’s. **a** depicts the cumulative number of differentially expressed genes (y-axis) as a mean with 95% CI (of 1000 times 200 random sampling) plotted against the *p* value used as cut-off to define differential expression (x-axis). **b** is a visualization of the genes that display significant differences between tumor stages in the whole group (ANOVA *p* < 0.05, *n* = 2764) using a t-SNE algorithm with clear separation of the immune (CMS1), mesenchymal (CMS4) and epithelial subtypes (CMS2/3). **c** displays the correlation between disseminated disease (stage III-IV) (x-axis) and CMS4 (y-axis) signature scores (r = 0.77, *p* < 0.001). **d** shows risk ratio’s for progression to advanced stages of disease (stage III-IV) and a 95% confidence interval comparing the different CMS subtypes

We tested the hypothesis that tumor stage as defined by TNM, does not only represent disease progression but also reflects different biological entities. By investigating the changes in the number of differentially expressed genes, considerable gene expression differences between TNM stages was revealed. These differences decreased significantly when stratified for CMS2 and CMS4 representing the most common CMSs (Fig. 2a). This was confirmed when stratifying for all subtypes (CMS1–4) (Additional file 1: Fig. S2). Furthermore, visualization of the genes that displayed significant differences between tumor stages (ANOVA *p* < 0.05, *n* = 2764) shows a clear separation for the immune (CMS1), epithelial (CMS2/3) and mesenchymal (CMS4) subtypes in both a t-SNE plot and a gene expression heatmap (Fig. 2b and Additional file 1: Fig. S3).

### CMS4 correlates with more advanced stages and has a higher progression rate

In order to specifically investigate the association between CMS4 and more advanced tumor stages, we built two gene signatures to discriminate disseminated disease (stage III-IV) from local disease (stage I-II), and to separate CMS4 cancers from CMS1/2/3 tumors (see methods). Remarkably, the two scores were highly correlated (r = 0.77, *p* < 0.001) (Fig. 2c), with only a few overlapping genes (13/200), suggesting that overrepresentation of CMS4 cancers in stage III-IV cancers is responsible for gene expression differences between early and advanced malignancies.

Subsequently, we assessed the rate of progression from early (stage I-II) to advanced (stage III-IV) tumor stage for each of the subtypes by calculating the risk ratios. This shows a markedly increased progression rate towards more advanced stages for CMS4 cancers as compared to CMS1 tumors (RR 1.64, 95% CI: 1.29–2.09), CMS2 (RR 1.25, 95% CI: 1.08–1.46) and CMS3 (RR 1.57, 95% CI: 1.23–2.01) (Fig. 2d).

### CMS4 holds prognostic value in high-risk stage II colon cancer

**Fig. 3:**
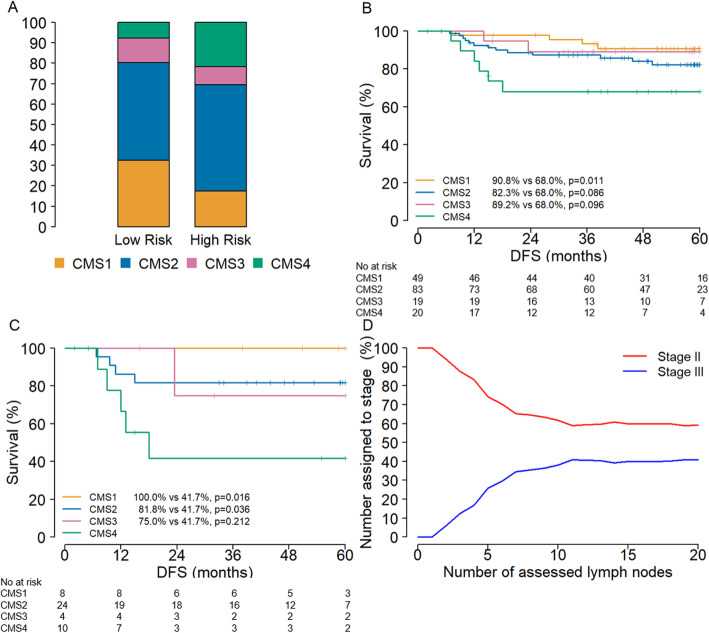
Lymph node assessment and disease free survival. **a** shows the distribution of CMS in stage II colon cancer (y-axis distribution in percentages) stratified for high-risk. **b** and **c** display the disease free survival (x-axis in months) of a set of patients with stage II colon cancer of the MATCH Cohort and GSE33113 (**b**), and the subset of patients with high-risk stage II colon cancer (T4 or < 10 assessed lymph nodes) (**c**). **d** shows that the chance of finding a positive lymph node (y-axis) increases with an increasing number of assessed lymph nodes (x-axis), which plateaus after 10 lymph nodes

In an effort to validate our findings and provide clinical utility to the insight obtained, we evaluated chemotherapy naive high-risk stage II colon cancers (Table 2). Based on the association between CMSs and tumor stage, we hypothesized that CMS4 cancers are over represented in high-risk stage II cancers. Indeed, in the combined stage II cohorts, MATCH and GSE33113 (*n* = 197), CMS4 cancers were more prevalent in high-risk stage II patients (21.7% vs 7.7%, *p* = 0.02 respectively) (Table 2, Fig. 3a and Additional file 1: Table S2). DFS for these patients confirmed the poor disease outcome of CMS4 cancers (Fig. 3b). This effect was explained by the poor outcome for patients with a CMS4 cancer in the subgroup with high-risk tumors (5-year DFS 41.7% versus 100.0%, *p* = 0.008) (Fig. 3c and Additional file 1: Fig. S4). These findings were substantiated by a multivariate analysis, which showed a significant correlation of CMS with DFS in the subgroup with high-risk tumors but not in the total stage II cohort (Table 3 and Additional file 1: Table S3). The extended GSE33113 cohort, comprising of both stage II and stage III tumors, revealed possible under-staging of high-risk stage II patients. With a rising number of assessed lymph nodes the percentage of stage III colon cancers increased (Fig. 3d and Additional file 1: Table S4).

**Table 2:** Characteristics MATCH and GSE33113

		Total		MATCH cohort		GSE33113	
		*n* = 197		*n* = 112		*n* = 85	
**Gender**	Female	101	51.3%	57	50.9%	44	51.8%
	Male	96	48.7%	55	49.1%	41	48.2%
**Age**	Median (IQR)	71.0 (63.0–77.0)		70.0 (63.0–76.0)		74.6 (61.9–80.2)	
T	3	184	93.4%	107	95.5%	77	90,6%
	4	13	6.6%	5	4.5%	8	9.4%
N	Median (range)	14	(1–46)	14	(5–28)	12	(1–46)
N	< 10 lymph nodes assesed	45	22.8%	14	12.5%	31	36.5%
	≥ 10 lymph nodes assesed	142	72.1%	98	87.5%	44	51.8%
	Missing	10	5.1%	0	0,0%	10	11.8%
**MSI**	MSS	140	71.1%	79	70.5%	61	71.8%
	MSI	52	26.4%	28	25.0%	24	28.2%
	Missing	5	2.5%	5	4.5%	0	0.0%
**CMS**	1	49	24.9%	29	25.9%	20	23.5%
	2	83	42.1%	52	46.4%	31	36.5%
	3	19	9.6%	11	9.8%	8	9.4%
	4	20	10.2%	5	4.5%	15	17,6%
	Indeterminate	26	13.2%	15	13.4%	11	12.9%

*IQR* Interquartile range

**Table 3:** Multivariate analysis of relevant parameters and disease-free survival for high-risk stage II patients

	HR	95% CI limits
CMS 1	^a^	
CMS 2	*0.225*	*0.053–0.957*
CMS 3	0.599	0.062–5.781
CMS 4	Reference	
Gender	2.725	0.488–15.225
Age	0.986	0.952–1.022
Location	3.45	0.799–14.85
T	2.006	0.360–11.173
MSI	^b^	

*CMS* Consensus molecular subtype, *MSI* Microsatellite instability

^a^Not estimable due to no events

^b^Not estimable due to no MSI patients

## Discussion

At present we are moving towards a more personalized medicine approach for the treatment of cancer. However, at this stage TNM staging is still the single most important feature guiding treatment decisions for CRC. The CMS classification is a promising classification system for CRC, identifying four subtypes with distinguishing biological features. CMS classification might be a relevant addition to TNM staging in order to provide an optimal treatment strategy for individual patients. Our findings support the hypothesis that tumor stage as defined by TNM, in addition to disease progression, resembles different biological entities. This adds to the argument that the CMS taxonomy is a potential framework to further tailor the prognostication and treatment of patients with CRC.

We have observed a difference in distribution for the CMS within the different TNM stages with mainly a decrease in CMS1 and a profound increase of CMS4 patients with advancing stages of disease. This is in line with the overall good prognosis of the CMS1, which are mainly MSI tumors, and the poor prognosis of the mesenchymal CMS4 subtype [4]. This may suggest that the poor prognosis for increased stages of disease is (in part) explained by the aggressive tumor biology of CMS4, given the poor disease outcome of CMS4 compared to CMS1–3 cancers. The aggressive nature of the mesenchymal subtype was also demonstrated by a higher progression rate for CMS4 compared to the other subtypes (Fig. 2d).

When stratified for CMSs, we observed a marked decrease in differentially expressed genes between the different tumor stages. Furthermore, a high correlation between the two gene signature scores for stage III/IV and CMS4 was demonstrated. This indicates that at least part of the biological differences between tumor stages are explained by the CMSs. Which in turn supports the hypothesis that different tumor stages are largely driven by tumor biology rather than disease progression.

Furthermore, we showed a possible and valuable clinical implication of the molecular subtypes for the high-risk stage II patients. Current guidelines recommend to consider adjuvant chemotherapy for these patients [8], which is based on literature showing (limited) prognostic value but no predictive value for the high-risk variables [9–13]. The overt difference in DFS for the CMS4 subtype in the subgroup of high-risk stage II patients suggests that CMS subtyping may be of added value to identify patients that have a high-risk, lymph node negative colon cancer. This effect might partly be explained by stage migration, due to under-staging as a result of low number of assessed lymph nodes; i.e. high-risk stage II tumors contain unrecognized stage III tumors. Another possible explanation for the marked difference in DFS within the CMS4 population is that these tumors behave more like the early-dissemination model [3, 14], instead of the classical linear-progression model in CRC. In agreement, the existence of early disseminating cancer cells which evolve independently at the metastatic site has been demonstrated in breast cancer [15]. Therefore CMS4 tumors may benefit from treatment with chemotherapy at an apparently early stage of progression (stage II).

Several clinical studies found that patients with synchronous and metachronous liver metastases had a similar overall survival upon diagnosis of metastatic disease [16–18]. This supports our hypothesis that tumor biology is installed at an early moment in tumor development and that this, rather than the progression over time, is the main determinant for prognosis in these patients. Also, determining the CMS may not only be helpful to identify high-risk stage II patients, but may also be used to select patients for specific treatments. Patients with an MSI tumor (mostly CMS1) are known to have very limited benefit from chemotherapy [19, 20]. However, these patients may very well benefit from immunotherapy or the addition of Bevacizumab instead of Cetuximab to chemotherapy in metastasized CRC [21, 22]. For epithelial-like tumors (CMS2/3) there is a predictive value for anti-EGFR therapy [7, 23]. Patients with a CMS2 tumor were shown to be responsive to Oxaliplatin-containing chemotherapy while mesenchymal tumors (CMS4) seemed refractory to 5FU-based chemotherapy. These results suggest that the CMS taxonomy may also be used to select patients for conventional chemotherapy [24, 25]. Future prospective studies should be conducted to confirm these hints on CMS-specific drug sensitivity, as these findings originate from retrospective studies.

The current study has several limitations. First, the survival analysis in the subset of stage II colon cancer may be subject to selection bias. Patients with high-risk stage II colon cancer were excluded from the current analysis when they did receive adjuvant chemotherapy. However, on estimate only 10–15% of these patients actually receive adjuvant chemotherapy, and patients with a T4 tumors and inadequate lymph node assessment (both high-risk factors) were present in the aggregated cohorts. Second, the additive value of the CMS for high-risk stage II patients should be validated in larger series given the relatively small number of patients in the high-risk stage II cohort.

## Conclusions

In conclusion, this study provides evidence to support the hypothesis that tumor stage and the corresponding prognosis are at least partly driven by tumor biology rather than the time of diagnosis. The CMS classification system has the potential to be a major contributor to clinical decision making. Therefore, future efforts should focus on further substantiating these findings and the development of a clinically applicable CMS test.

## Supplementary information

**Additional file 1: Supplementary Table S1.** Distribution of CMS per tumor stage in the total and individual cohorts. **Supplementary Figure S1.** Distribution of the molecular subtypes per tumor stage in the individual cohorts. **Supplementary Figure S2.** Random sampling all subtypes *n* = 130. **Supplementary Figure S3.** Heatmap of the differentially expressed genes between tumor stages. **Supplementary Table S2.** Distribution of the molecular subtypes in high and low risk stage II CRC patients. **Supplementary Figure S4.** Disease-free survival in patients with ≥ 10 lymph nodes assessed. **Supplementary Table S3.** Multivariate analysis of CMS and disease free survival for total stage II cohort. **Supplementary Table S4.** Characteristics extended GSE33113 cohort.

## Data Availability

The GSE39582 [1], GSE33113 [2] and TCGA dataset are publicly available in the Gene Expression Omnibus repository (https://www.ncbi.nlm.nih.gov/geo/) and the TCGA repository (https://cancergenome.nih.gov/). The sequencing data and a restricted clinical data set of the MATCH cohort can be accessed through the European Genome Phenome Archive (https://www.ebi.ac.uk/ega/home) under accession number EGAS00001002197 and as supplemental data of Kloosterman et al. [3] Detailed clinical data of the MATCH Cohort and the data set of the extended AMC-AJCCII-90 Cohort will be provided upon reasonable request to the corresponding author.

## Abbreviations

CMS: Consensus Molecular Subtype
CRC: Colorectal Cancer
MSI: Microsatellite Instability
