# Supplementary material for: Interconnectivity between molecular subtypes and tumor stage in colorectal cancer
Source: BMC Cancer. 2020 Sep 4;20:850. doi: 10.1186/s12885-020-07316-z (PMC7473811; doi:10.1186/s12885-020-07316-z)
Supplement: Supplementary file 1 — Additional file 1: Supplementary Table S1. Distribution of CMS per tumor stage in the total and individual cohorts. Supplementary Figure S1. Distribution of the molecular subtypes per tumor stage in the individual cohorts. Supplementary Figure S2. Random sampling all subtypes n = 130. Supplementary Figure S3. Heatmap of the differentially expressed genes between tumor stages. Supplementary Table S2. Distribution of the molecular subtypes in high and low risk stage II CRC patients. Supplementary Figure S4. Disease-free survival in patients with ≥ 10 lymph nodes assessed. Supplementary Table S3. Multivariate analysis of CMS and disease free survival for total stage II cohort. Supplementary Table S4. Characteristics extended GSE33113 cohort. [file 12885_2020_7316_MOESM1_ESM.docx]

**Supplementary Table S1.** Distribution of CMS per tumor stage in the total and individual cohorts.

**Total cohort** (n=94 mixed/ indeterminate)

|  |  |  |  |  |  |  |  |
| --- | --- | --- | --- | --- | --- | --- | --- |
|  |  | **Stage I** | **Stage II** | **Stage III** | **Stage IV** |  | **Total** |
|  |  |  |  |  |  |  |  |
| **CMS 1** |  | 21 | 78 | 46 | 8 |  | 153 |
|  |  | 17.2% | 20.1% | 14.4% | 6.8% |  |  |
| **CMS 2** |  | 63 | 163 | 141 | 53 |  | 420 |
|  |  | 51.6% | 42.0% | 44.2% | 45.3% |  |  |
| **CMS 3** |  | 26 | 58 | 38 | 11 |  | 133 |
|  |  | 21.3% | 14.9% | 11.9% | 9.4% |  |  |
| **CMS 4** |  | 12 | 89 | 94 | 45 |  | 240 |
|  |  | 9.8% | 22.9% | 29.5% | 38.5% |  |  |
|  |  |  |  |  |  |  |  |
| **Total** |  | 122 | 388 | 319 | 117 |  | 946 |
|  |  | 100.0% | 100.0% | 100.0% | 100.0% |  | 100.0% |
|  |  |  |  |  |  |  |  |

**GSE39582** (n=40 mixed/ indeterminate)

|  |  |  |  |  |  |  |  |
| --- | --- | --- | --- | --- | --- | --- | --- |
|  |  | **Stage I** | **Stage II** | **Stage III** | **Stage IV** |  | **Total** |
|  |  |  |  |  |  |  |  |
| **CMS 1** |  | 8 | 37 | 30 | 4 |  | 79 |
|  |  | 22.9% | 18.3% | 16.5% | 7.7% |  | 16.8% |
| **CMS 2** |  | 17 | 97 | 81 | 19 |  | 214 |
|  |  | 48.6% | 48.0% | 44.5% | 36.5% |  | 45.4% |
| **CMS 3** |  | 7 | 29 | 23 | 7 |  | 66 |
|  |  | 20.0% | 14.4% | 12.6% | 13.5% |  | 14.0% |
| **CMS 4** |  | 3 | 39 | 48 | 22 |  | 112 |
|  |  | 8.6% | 19.3% | 26.4% | 42.3% |  | 23.8% |
|  |  |  |  |  |  |  |  |
| **Total** |  | 35 | 202 | 182 | 52 |  | 471 |
|  |  | 100.0% | 100.0% | 100.0% | 100.0% |  | 100.0% |
|  |  |  |  |  |  |  |  |

**TCGA** (n=54 mixed/ indeterminate)

|  |  |  |  |  |  |  |  |
| --- | --- | --- | --- | --- | --- | --- | --- |
|  |  | **Stage I** | **Stage II** | **Stage III** | **Stage IV** |  | **Total** |
|  |  |  |  |  |  |  |  |
| **CMS 1** |  | 13 | 41 | 16 | 4 |  | 74 |
|  |  | 14.9% | 22.0% | 11.7% | 6.2% |  | 15.6% |
| **CMS 2** |  | 46 | 66 | 60 | 34 |  | 206 |
|  |  | 52.9% | 35.5% | 43.8% | 52.3% |  | 43.4% |
| **CMS 3** |  | 19 | 29 | 15 | 4 |  | 67 |
|  |  | 21.8% | 15.6% | 10.9% | 6.2% |  | 14.1% |
| **CMS 4** |  | 9 | 50 | 46 | 23 |  | 128 |
|  |  | 10.3% | 26.8% | 33.6% | 35.4 |  | 26.9% |
|  |  |  |  |  |  |  |  |
| **Total** |  | 87 | 186 | 137 | 65 |  | 475 |
|  |  | 100.0% | 100.0% | 100.0% | 100.0% |  | 100.0% |
|  |  |  |  |  |  |  |  |

CMS, consensus molecular subtype

**Supplementary Figure S1.** Distribution of the molecular subtypes per tumor stage in the individual cohorts.


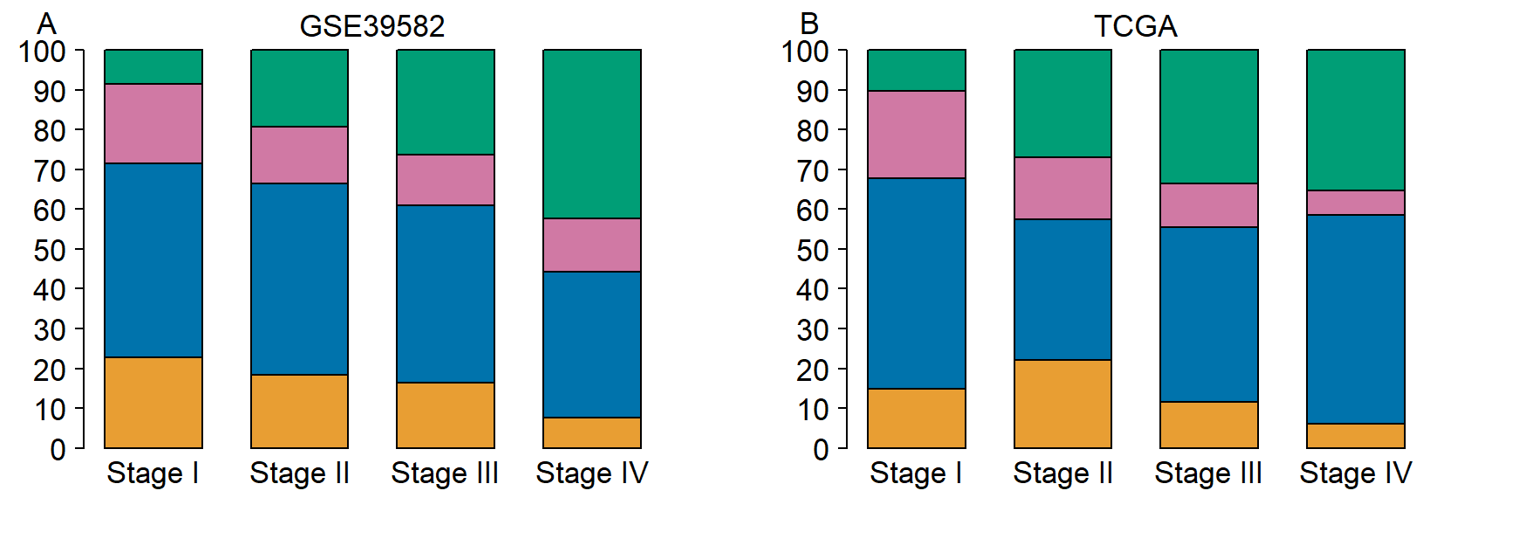


**Supplementary Figure S2. Random sampling all subytpes n=130.**

The cumulative number of differentially expressed genes (y-axis) as a mean with 95% CI (of 1000 times 130 random sampling) plotted against the p value used as cut-off to define differential expression (x-axis). A random sampling of 130 instead of 200 of the main figure 2A was used due to a low number of cases for CMS1 and CMS3.


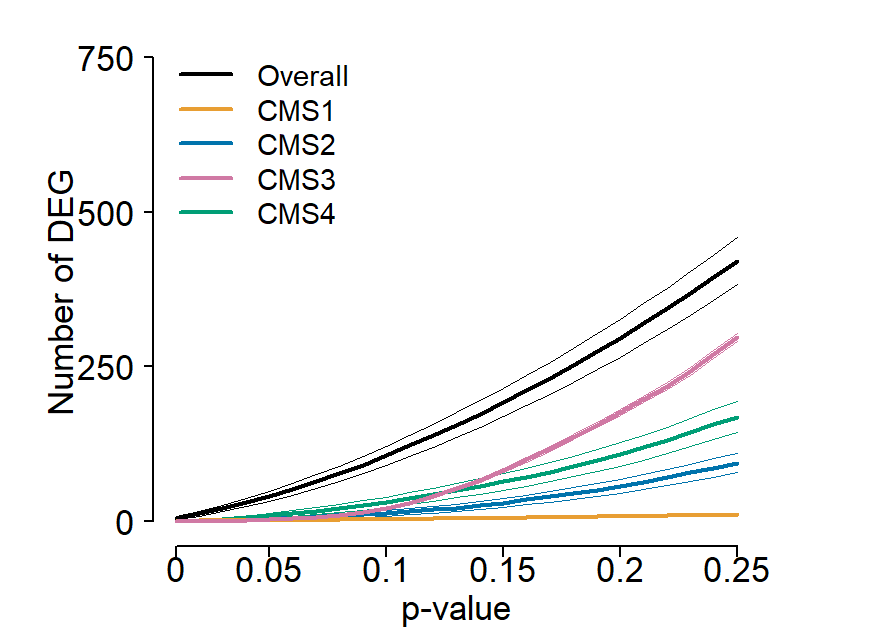


**
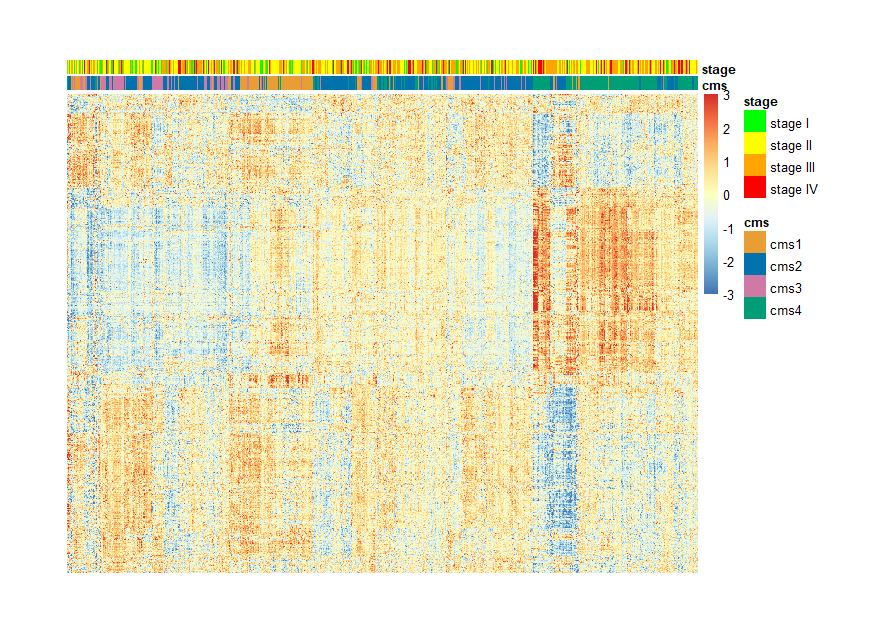
Supplementary Figure S3.** Heatmap of the differentially expressed genes between tumor stages.

A heatmap of the genes that display significant differences between tumor stages in the whole group (ANOVA p<0.05, n=2384) showing a clear separation of the mesenchymal (CMS4) and the epithelial subtypes (CMS2/3). Columns and rows were hierarchically clustered.

**Supplementary Table S2.** Distribution of the molecular subtypes in high and low risk stage II CRC patients.

|  |  |  |  |
| --- | --- | --- | --- |
|  |  | **Low Risk** | **High Risk** |
|  |  |  |  |
| **CMS 1** |  | 38 | 8 |
|  |  | 32.5% | 17.4% |
| **CMS 2** |  | 56 | 24 |
|  |  | 47.9% | 52.2% |
| **CMS 3** |  | 14 | 4 |
|  |  | 12.0% | 8.7% |
| **CMS 4** |  | 9 | 10 |
|  |  | 7.7% | 21.7% |
|  |  |  |  |

CMS, consensus molecular subtype

**Supplementary Figure S4.** Disease-free survival in patients with ≥ 10 lymph nodes assessed.

The poor disease free survival for stage II CRC with a CMS4 tumor (Figure 3A) was explained by the poor outcome for patients with inadequate lymph node assessment (Figure 3B) and not by patients with adequate lymph node assessment as shown in this figure.


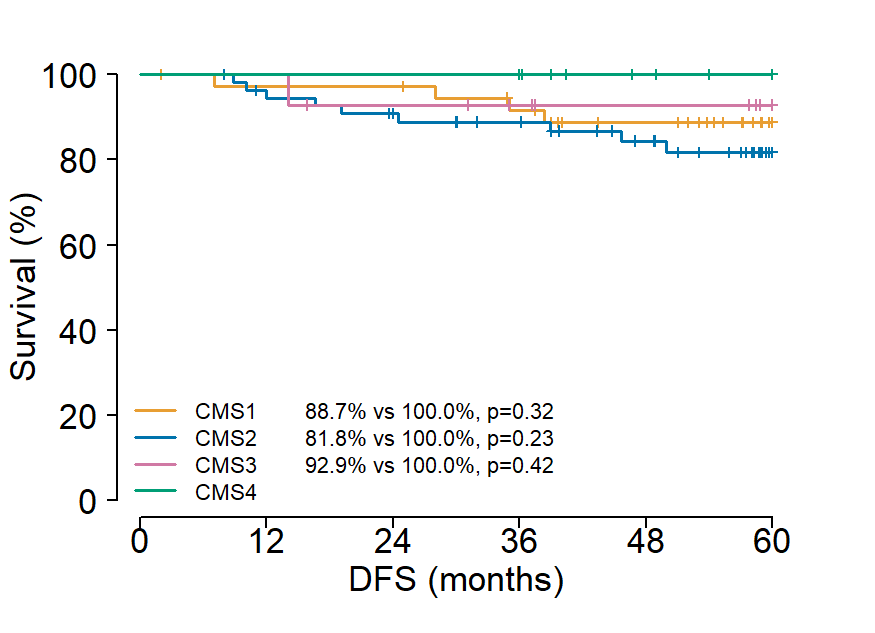

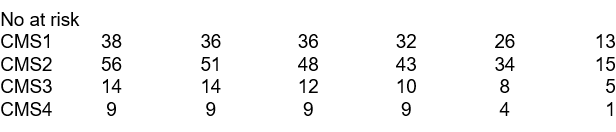


**Supplementary Table S3.** Multivariate analysis of CMS and disease free survival for total stage II cohort

|  | **HR** | **95% CI limits** |
| --- | --- | --- |
| CMS 1 | 0.387 | 0.065-2.282 |
| CMS 2 | 0.459 | 0.169-1.248 |
| CMS 3 | 0.325 | 0.064-1.655 |
| CMS 4 | Reference |  |
| Gender | 1.791 | 0.767-4.179 |
| Age | 0.995 | 0.963-1.028 |
| Location | 1.188 | 0.490-2.883 |
| T | 2.176 | 0.469-10.089 |
| MSI | 0.475 | 0.080-2.818 |

CMS, consensus molecular subtype; MSI, microsatellite instability

**Supplementary Table S4.** Characteristics extended GSE33113 cohort.

|  |  |  |  |
| --- | --- | --- | --- |
|  |  | **Total** |  |
|  |  | n=410 |  |
|  |  |  |  |
| **Gender** | Female | 205 | 50% |
|  | Male | 205 | 50% |
|  |  |  |  |
| **Age** | median (IQR^a^) | 68 | (59-77) |
|  |  |  |  |
| TNM | 2  3 | 246  164 | 60%  40% |
|  |  |  |  |
| Ln Assesed | median (range) | 11 | (0-100) |
|  |  |  |  |
| Ln Assessed | < 10 | 159 | 39% |
|  | ≥ 10 | 225 | 55% |
|  | missing | 26 | 6% |
|  |  |  |  |
| **Tumor location** | Left | 250 | 61% |
|  | Right | 155 | 38% |
|  | Both | 5 | 1% |

^a^IQR = interquartile range
